# Cancer and Other Tumours of the Stomach

**Published:** 1903-09

**Authors:** 


**Cancer and other Tumours of the Stomach.** By SAMUEL FENWICK, M.D., and W. SOLTAU FENWICK, M.D. Pp. xv., 362. London: J. and A. Churchill. 1902.

This is an excellent monograph on an important subject, and every detail of it is adequately dealt with. The section on Etiology is especially interesting, as the authors adduce good evidence for some opinions that are not in accordance with generally accepted views. Thus they give the incidence on sex as about equal, and show that "the tendency to gastric carcinoma steadily increases with each decade of life until the age of 75." They also show that the mortality from cancer of the stomach is higher in the summer than in the winter. The account of the symptomatology is clear, and gives an accurate picture of the disease, and that of the physical signs is also good. We think, however, fuller directions for the examination of the gastric contents should have been given; but the authors may perhaps have considered that as they were dealing with one variety of stomach disorder, it was not necessary to give more than those tests which are specially useful in this particular disease. A very useful section, considering the protean forms under which cancer of the stomach may appear, deals with the clinical varieties of the disease, and this has been wisely illustrated by a few well-selected, and not too lengthy, descriptions of cases. The advice upon treatment and the chance of success given by surgical interference in the different forms of the disease is distinguished by good sense and sound judgment. The second part of the work deals with Tumours of the Duodenum, Syphilis, Cysts, and Benign Tumours of the Stomach, and in this more recondite subject the authors are equally at home. Cancer of the stomach is unfortunately so frequent, and, as we have said, shows itself under such varying aspects, that it is most important that we should all be thoroughly acquainted with the subject, and in this book the practitioner will find a guide on which he can safely rely. The book is well illustrated and printed.

